# Molecular Determinants of Sulfadoxine-Pyrimethamine Resistance in Plasmodium falciparum Isolates from Central Africa between 2016 and 2021: Wide Geographic Spread of Highly Mutated *Pfdhfr* and *Pfdhps* Alleles

**DOI:** 10.1128/spectrum.02005-22

**Published:** 2022-09-19

**Authors:** Xiaoxiao Wang, Xuan Zhang, Hualiang Chen, Qiaoyi Lu, Wei Ruan, Zhiping Chen

**Affiliations:** a Zhejiang Provincial Center for Disease Control and Prevention, Zhejiang, People’s Republic of China; Hebrew University of Jerusalem

**Keywords:** molecular determinants, *Plasmodium falciparum*, resistance

## Abstract

Sulfadoxine-pyrimethamine (SP) resistance impairs the efficacy of antimalarial drugs. Monitoring molecular markers in exported malaria infections provides an efficient way to trace the emergence of drug resistance in countries where malaria is endemic. Molecular markers in *Pfdhfr* and *Pfdhps* of 237 Plasmodium falciparum infections imported from central Africa between 2016 and 2021 were detected. The spatial and temporal distributions of *Pfdhfr* and *Pfdhps* mutations were analyzed. A high prevalence of *Pfdhfr* single-nucleotide polymorphisms (SNPs) (~92.34% to 99.10%) and a high frequency of the triple mutation haplotype **I**_51_**R**_59_**N**_108_ were observed. Cameroon, Equatorial Guinea, and Gabon showed a higher frequency (~96.61% to 100.00%) of **I**_51_**R**_59_**N**_108_ than other countries (~71.11% to 88.10%). The prevalence of C59R and **I**_51_**R**_59_**N**_108_ increased while that of other SNPs or haplotypes did not fluctuate greatly from 2016 to 2021. Large proportions of *Pfdhps* SNPs (A437G and K540E) were demonstrated. The SNP distribution of *Pfdhps* differed between countries, with S436A dominating in northern countries and A437G dominating in others. The proportions of I431V, A437G, and the triple mutant haplotype declined between 2016 and 2021, whereas the prevalence of the single mutant haplotype rose from 61.60% to 73.68%. Combinations of *Pfdhfr*-*Pfdhps* alleles conferring partial resistance, full resistance, and superresistance to SP, as defined in the text, were detected in 63.64%, 8.64%, and 0.91% of the samples, respectively. The octuple *Pfdhfr*-*Pfdhps* allele (**I**_51_**R**_59_**N**_108_-**V**_431_**A**_436_**G**_437_K_540_**G**_581_**S**_613_) was seen in 5.00% of the samples. We demonstrated the wide geographic spread and increasing trends in highly SP-resistant *Pfdhfr* genes and varying spatial patterns of *Pfdhps* mutants across countries in central Africa. The high prevalences of partially resistant, fully resistant, and superresistant *Pfdhfr-Pfdhps* combinations observed here indicated impaired SP efficacy. Increased molecular surveillance is required to monitor the changing status of the *Pfdhfr* and *Pfdhps* genes.

**IMPORTANCE** Monitoring drug resistance is important for malaria control because its early detection enables timely action to prevent its spread and mitigate its impact. The wide geographic spread and the increasing trend of highly resistant *Pfdhfr* genes between 2016 and 2021 found in our study are worrisome and emphasize the urgency to monitor their updated status in central Africa. This study also illustrated the wide spread of the novel mutant *Pfdhps* I431V as well as the high prevalence of “partially resistant,” “fully resistant,” and “superresistant” *Pfdhfr*-*Pfdhps* combinations, indicating the urgent concern for SP efficacy in central Africa. These findings are alarming in central African countries where malaria is endemic, where SP was is widely used for the intermittent preventive treatment of malaria in pregnancy (IPTp) and the intermittent preventive treatment of malaria in infants below 5 years of age (IPTi), and urge enhanced molecular surveillance and responses to the threat of drug resistance.

## INTRODUCTION

Malaria is a potentially lethal disease that threatened nearly half of the world’s population in 2020 (1). The World Health Organization (WHO)-designated African region bears 95% of patients and 96% of deaths. Central Africa, including 10 countries located at the center of the WHO African region, accounted for nearly 25% of the estimated cases of malaria in the WHO African region in 2020 (1). It was estimated that more than 54 million infections and almost 140,100 deaths occurred in central Africa in 2020 (1).

Antimalarial drugs must remain efficacious to save the lives of millions of people with malaria infection. Sulfadoxine and pyrimethamine (SP) act primarily on the enzymes dihydropteroate synthase and dihydrofolate reductase, respectively, which are necessary for folate biosynthesis by the parasite (2). Because they synergistically affect the same biochemical pathway, these two drugs are generally used in combination and are referred to as monotherapy. Although the SP combination has been removed from the list of first-line treatments for malaria in the majority of African countries since 2008 (3), it is currently used for the intermittent preventive treatment of malaria in pregnancy (IPTp) and the intermittent preventive treatment of malaria in infants below 5 years of age (IPTi) in most central African countries to protect mothers during pregnancy and to protect against the consequences of malaria in newborns (1, 4–8).

However, the emergence of resistance to antimalarial drugs is a significant obstacle to reducing the burden of this disease (9, 10). Monitoring drug resistance is important because its early detection allows timely action to be taken to prevent its spread and mitigate its impact on global health. The WHO’s 2016–2030 global technical strategy for malaria suggests monitoring the efficacy of antimalarial medicines in countries where malaria is endemic (11). Several tools can be used to assess drug resistance (12, 13). In the past, therapeutic efficacy studies were regarded as the gold-standard method for tracking clinical and parasitological outcomes, and integrated drug efficacy surveillance was recommended in low-transmission countries where each case can be effectively managed. However, molecular marker studies, which determine the prevalences and patterns of key molecular mutations, have become the most widely used methods because of their advantages of simple molecular analysis and the convenient transport and storage of blood samples (12).

SP resistance occurs via point mutations in the *Plasmodium Pfdhfr* and *Pfdhps* genes that result in substitutions in the SP-targeting enzymes dihydrofolate reductase and dihydropteroate synthetase, respectively (14–16). SP resistance depends on the numbers of mutations in the *Pfdhfr* and *Pfdhps* genes. Combinations of mutant *Pfdhfr* and *Pfdhps* genes have been classified into three types by Naidoo and Roper (17): the combination of N51I, N59R, and S108N in *Pfdhfr* and A437G in *Pfdhps*, conferring partial resistance; the combination of *Pfdhfr* N51I, N59R, and S108N and *Pfdhps* A437G and K540E, conferring full resistance; and *Pfdhps* A581G in addition to the five major mutations described above, conferring superresistance to SP. The fully resistant type, a combination of the double mutant *Pfdhfr*(A437G,K540E) and the triple mutant *Pfdhps*(N51I,C59R,S108N), is associated with the failure of clinical SP treatment (17, 18).

Surveillance of the molecular markers associated with drug resistance among patients with imported malaria should allow the spread of resistant parasites to be assessed (15). Three countries in central Africa account for more than 80% of the estimated infections in that region: the Democratic Republic of the Congo (Congo DR) accounts for 53.1% of the estimated cases, followed by Angola (15.1%) and Cameroon (12.6%) (1). Plasmodium falciparum infections are imported from central Africa to Zhejiang Province, China, from countries with a similar distribution (mainly from Congo DR, Angola, and Cameroon), suggesting that the imported malaria cases in Zhejiang Province are a representative subset of those in central Africa. Monitoring molecular markers in patients with imported malaria provides an efficient way to trace the emergence of drug resistance in countries where malaria is endemic, particularly in those where field surveillance is difficult. Here, we collected samples from patients with malaria imported from central Africa to Zhejiang Province, China, between 2016 and 2021 and investigated the genetic mutations in the *Pfdhfr* and *Pfdhps* genes associated with resistance to antimalarial drugs. Our purpose was to better understand the status and spectrum of drug resistance of *Plasmodium* parasites in central Africa by examining their molecular epidemiology to gather evidence to guide drug policy updates in the study area.

## RESULTS

### General information.

**FIG 1:**
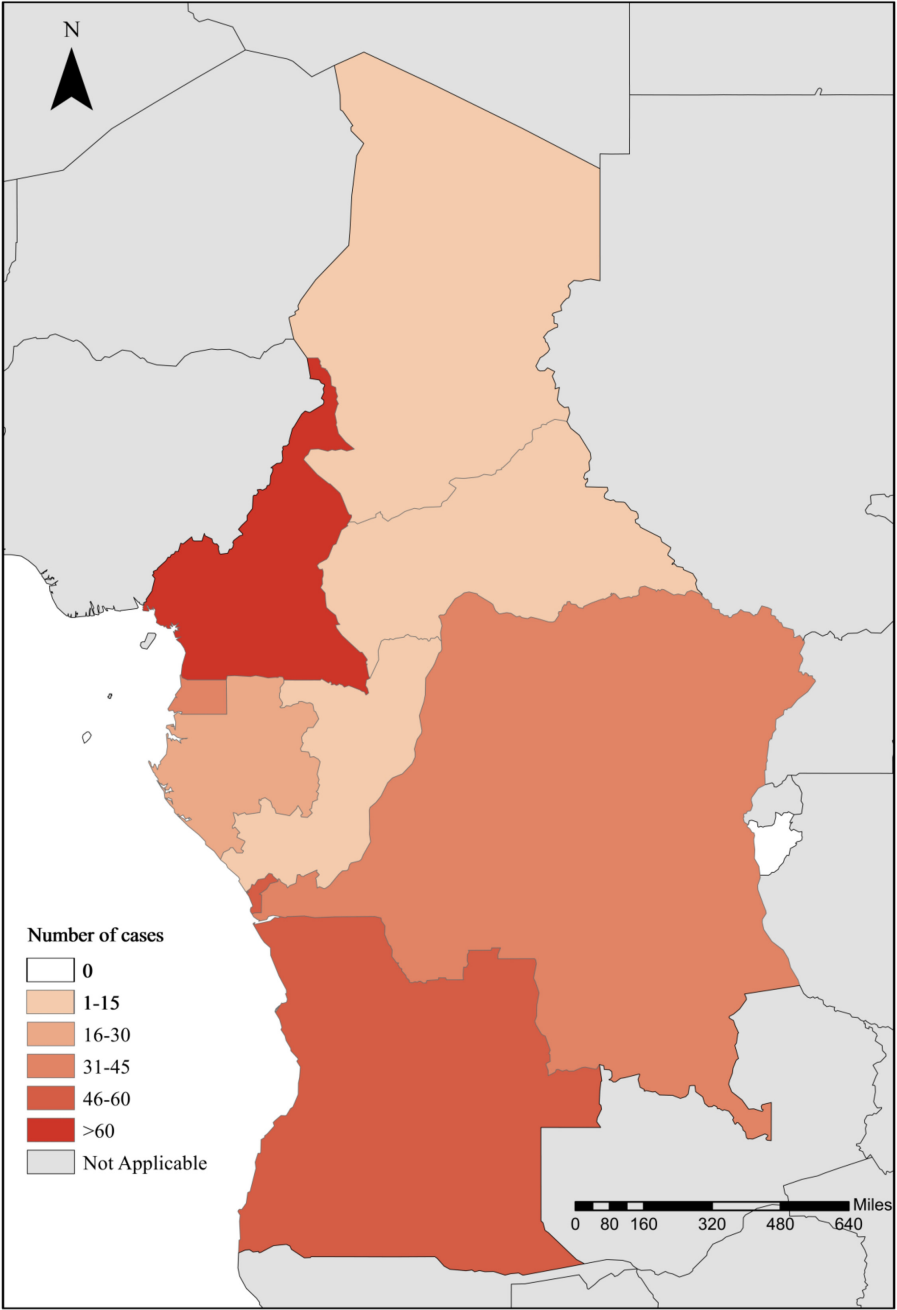
Number of P. falciparum infections imported from central African countries.

**FIG 2:**
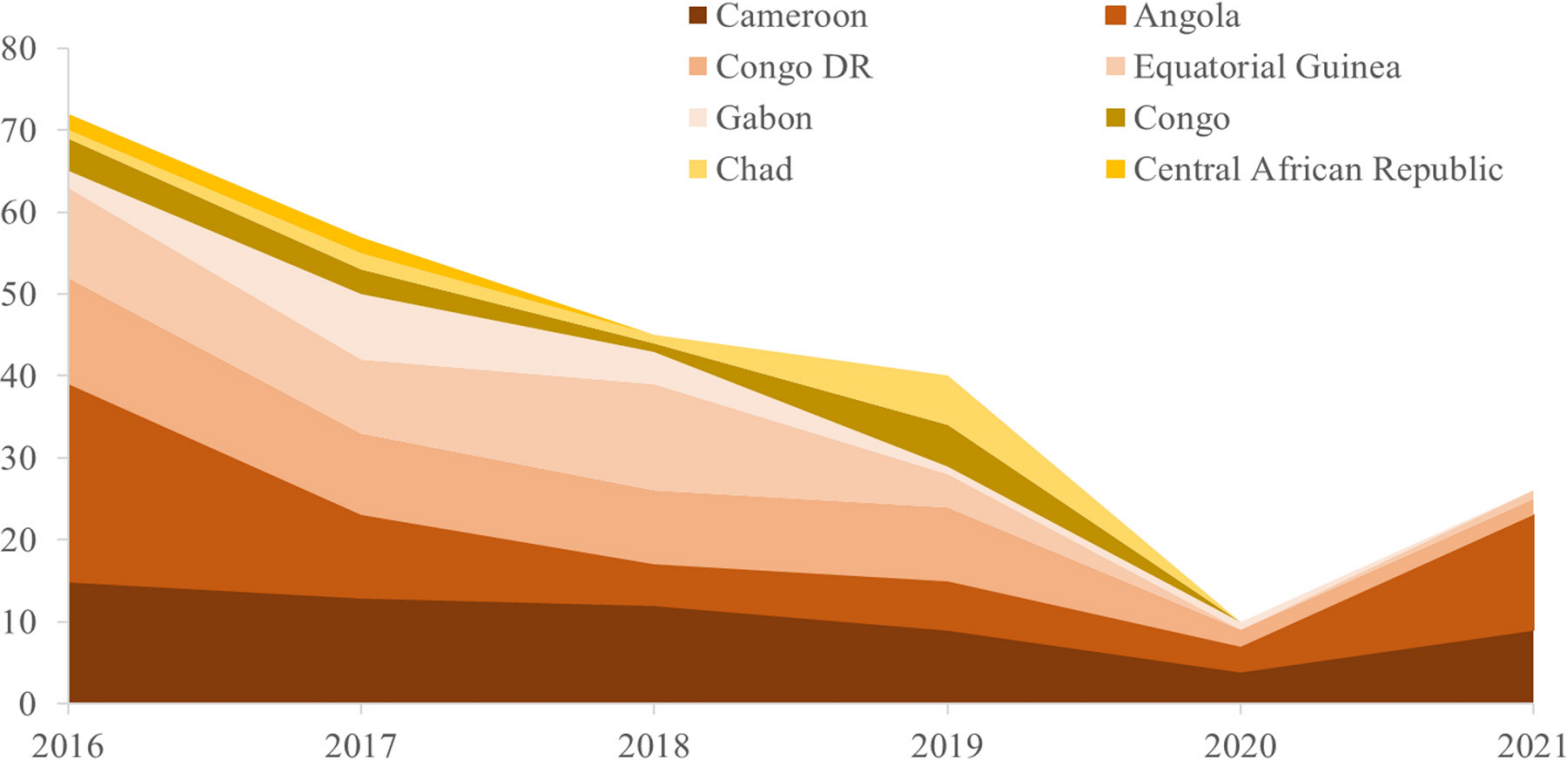
Temporal distribution of P. falciparum cases imported from central African countries between 2016 and 2021.

A total of 237 P. falciparum infections were imported from central African countries to Zhejiang Province, China, between 2016 and 2021. Patients with malaria traveled back from 8 of the 10 countries in central Africa. Four countries in the region accounted for more than 80% of the imported patients: Cameroon (26.16%), Angola (20.68%), Congo DR (18.99%), and Equatorial Guinea (16.03%) (Fig. 1 and Table 1). In terms of the temporal distribution of cases, a sharp reduction was seen from 2016 to 2021 (Fig. 2), particularly between 2020 and 2021, which may have resulted from the coronavirus disease 2019 (COVID-19) pandemic. The number of imported cases declined from 72 in 2016 to 10 in 2020 and 13 in 2021 because of travel restrictions in the age of COVID-19. Especially, no cases were imported from Congo, Chad, and the Central African Republic between 2020 and 2021. Of the 237 cases detected, 208 (87.76%) were male patients. The median age (range) was 44 (18 to 66) years.

**TABLE 1:** Temporal and spatial distribution of P. falciparum cases imported from central Africa to Zhejiang Province between 2016 and 2021

Country	No. of cases by yr						Total no. of cases (%)
	2016	2017	2018	2019	2020	2021	
Cameroon	15	13	12	9	4	9	62 (26.16)
Angola	24	10	5	6	3	14	49 (20.68)
Congo DR	13	10	9	9	2	2	45 (18.99)
Equatorial Guinea	11	9	13	4	0	1	38 (16.03)
Gabon	2	8	4	1	1	0	16 (6.75)
Congo	4	3	1	5	0	0	13 (5.49)
Chad	1	2	1	6	0	0	10 (4.22)
Central African Republic	2	2	0	0	0	0	4 (1.69)

Total	72	57	45	40	10	13	237 (100.00)

### Spatial distribution of SNPs and haplotypes of *Pfdhfr*.

**FIG 3:**
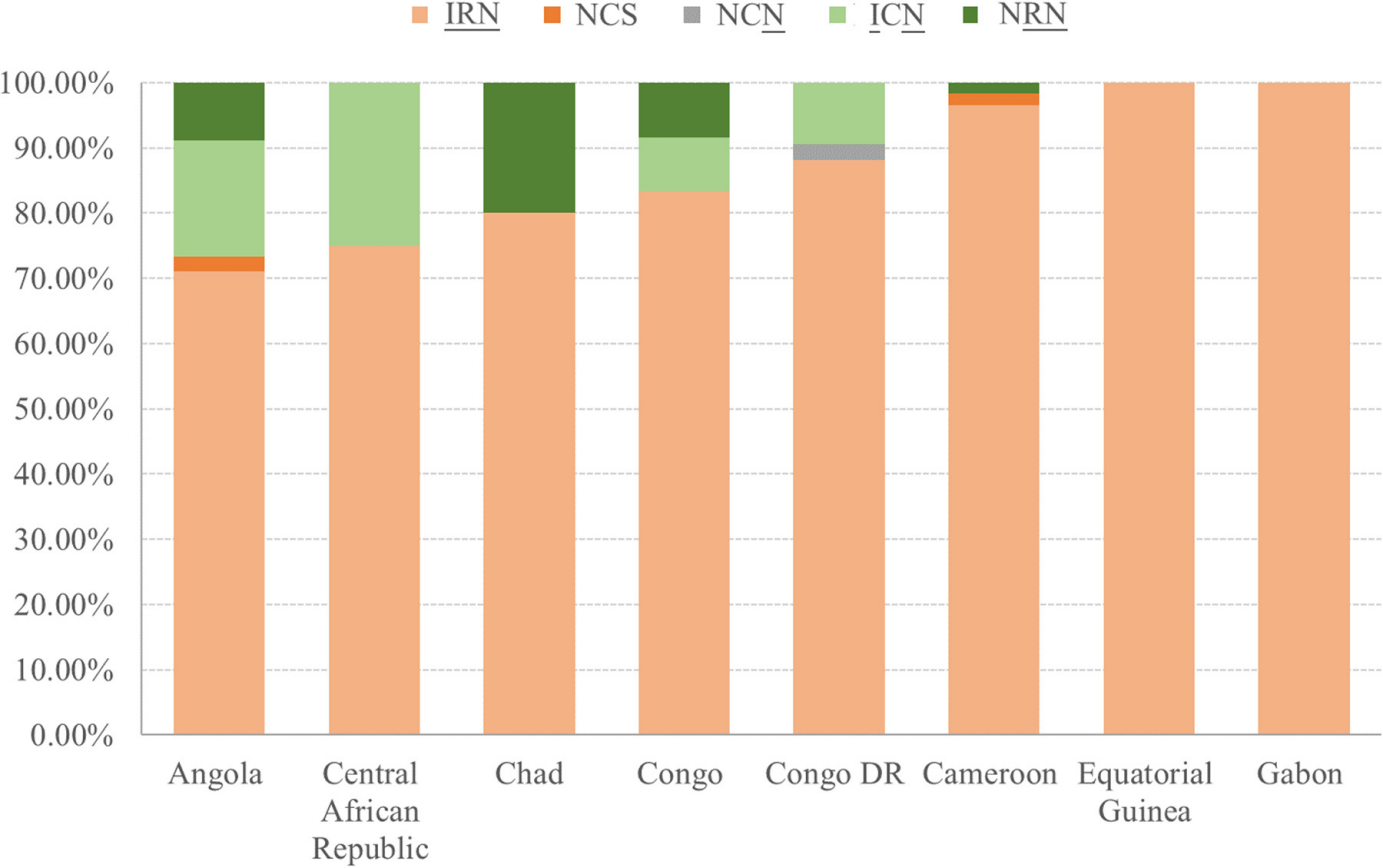
Spatial distribution of *Pfdhfr* genotypes in P. falciparum cases imported from central African countries between 2016 and 2021. NCS, N_51_C_59_S_108_; NCN, N_51_C_59_**N**_108_; ICN, **I**_51_C_59_**N**_108_; NRN, N_51_**R**_59_**N**_108_; IRN, **I**_51_**R**_59_**N**_108_.

A total of 222 sequences of *Pfdhfr* were successfully obtained from 237 samples. Sequence alignment revealed the high prevalence of the single-nucleotide polymorphisms (SNPs) N51I (95.05%; 211/222), C59R (92.34%; 205/222), and S108N (99.10%; 220/222) in all of the central African countries (Table 2). In particular, all of the isolates carried the S108N mutation, except for one sample from Cameroon. The point mutations C50R, D139V, and I164L were not found. A total of five haplotypes were identified, including wild-type haplotype N_51_C_59_S_108_, single mutant type N_51_C_59_**N**_108_ (boldface type indicates the mutant amino acid), double mutant types **I**_51_C_59_**N**_108_ and N_51_**R**_59_**N**_108_, and triple mutant type **I**_51_**R**_59_**N**_108_. Because the mutant SNPs N51I, C59R, and S108N were very frequent, the triple mutant **I**_51_**R**_59_**N**_108_ was the most frequent allele (88.74%; 197/222) in all of the sampled countries (Fig. 3). Wild-type haplotype N_51_C_59_S_108_ (0.90%; 2/222) and single mutant type N_51_C_59_**N**_108_ (0.45%; 1/222) were seldom observed. Notably, all of the parasite strains from Equatorial Guinea and Gabon carried the triple mutation **I**_51_**R**_59_**N**_108_. The double mutants **I**_51_C_59_**N**_108_ and N_51_**R**_59_**N**_108_ were also seen in some isolates (6.31% and 3.60%, respectively).

**TABLE 2:** Polymorphisms observed in *Pfdhfr* from P. falciparum isolates imported from central Africa

Country	No. of samples	No. of isolates with SNP (%)			No. of isolates with haplotype (%)				
		N51I	C59R	S108N	N_51_C_59_S_108_	N_51_C_59_N_108_	I_51_C_59_N_108_	N_51_R_59_N_108_	I_51_R_59_N_108_
Cameroon	59	57 (96.61)	58 (98.31)	58 (98.31)	1 (1.69)	0	0 (0)	1 (1.69)	57 (96.61)
Angola	45	40 (88.89)	36 (80.00)	44 (97.78)	1 (2.22)	0	8 (17.78)	4 (8.89)	32 (71.11)
Congo DR	42	41 (97.62)	37 (88.10)	42 (100.00)	0	1 (2.38)	4 (9.52)	0	37 (88.10)
Equatorial Guinea	36	36 (100.00)	36 (100.00)	36 (100.00)	0	0	0	0	36 (100.00)
Gabon	14	14 (100.00)	14 (100.00)	14 (100.00)	0	0	0	0	14 (100.00)
Congo	12	11 (91.67)	11 (91.67)	12 (100.00)	0	0	1 (8.33)	1 (8.33)	10 (83.33)
Chad	10	8 (80.00)	10 (100.00)	10 (100.00)	0	0	0 (0)	2 (22.00)	8 (80.00)
Central African Republic	4	4 (100.00)	3 (75.00)	4 (100.00)	0	0	1 (25.00)	0	3 (75.00)

Total	222	211 (95.05)	205 (92.34)	220 (99.10)	2 (0.90)	1 (0.45)	14 (6.31)	8 (3.60)	197 (88.74)

### Temporal distribution of SNPs and haplotypes of *Pfdhfr*.

**FIG 4:**
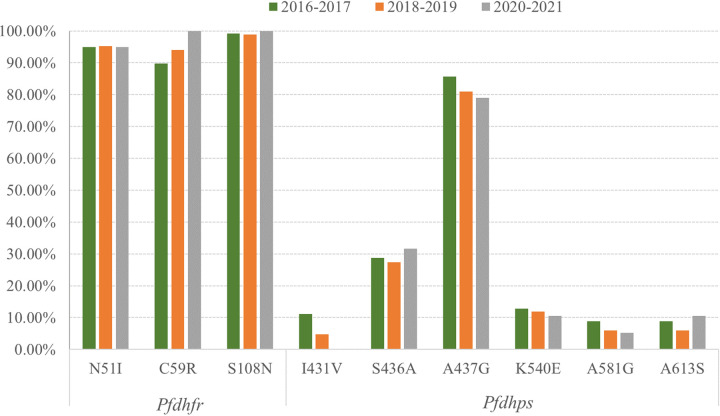
Temporal distribution of SNPs in *Pfdhfr* and *Pfdhps* in P. falciparum cases imported from central African countries between 2016 and 2021.

**FIG 5:**
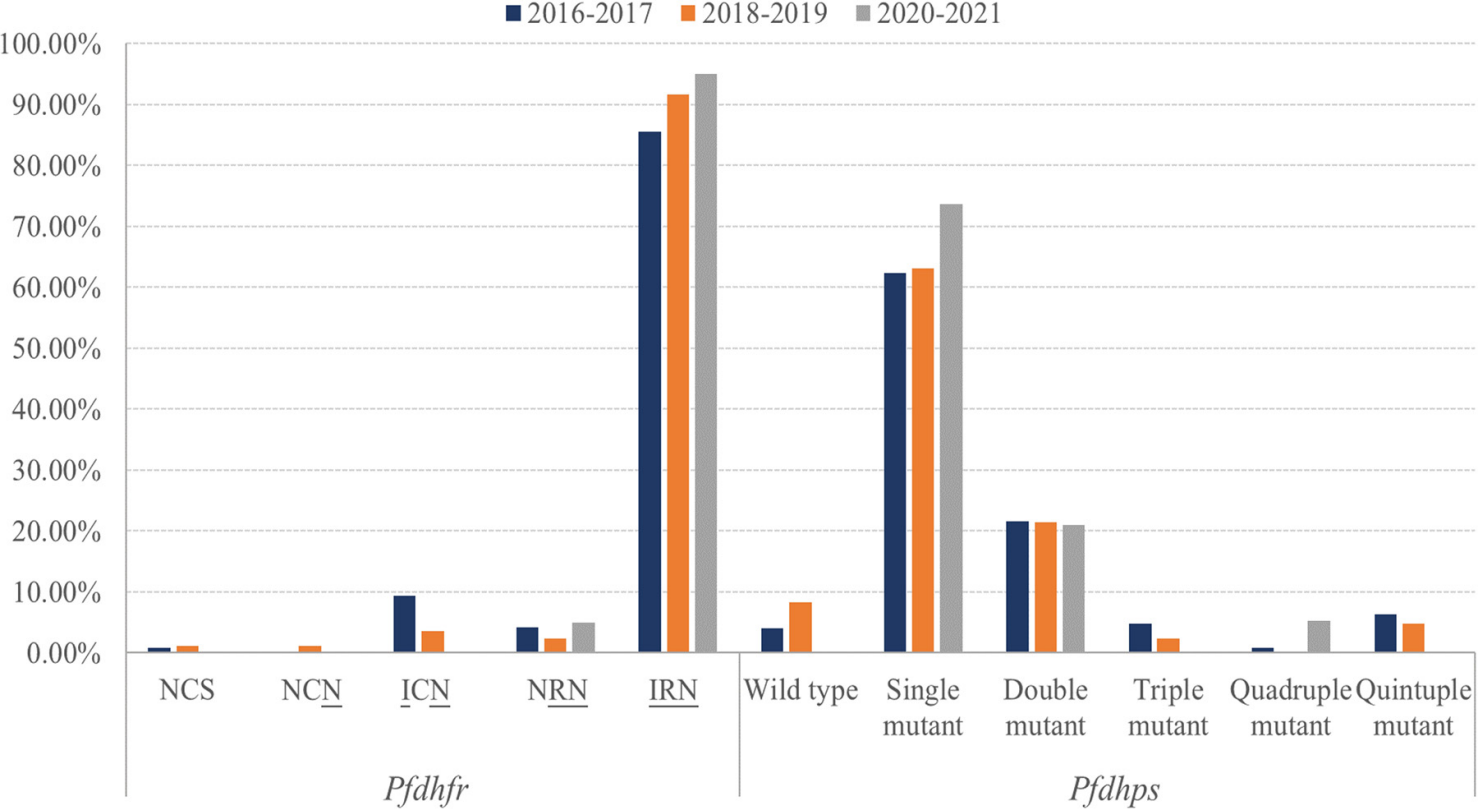
Temporal distribution of haplotypes of *Pfdhfr* and *Pfdhps* in P. falciparum cases imported from central African countries between 2016 and 2021. NCS, N_51_C_59_S_108_; NCN, N_51_C_59_**N**_108_; ICN, **I**_51_C_59_**N**_108_; NRN, N_51_**R**_59_**N**_108_; IRN, **I**_51_**R**_59_**N**_108_.

An increase in the frequency of C59R was observed between 2016 and 2021, although it was not statistically significant (χ^2^ for trend = 3.003; *P* = 0.383). Specifically, the proportion of samples with C59R was 89.93% in 2016 to 2017, which climbed to 94.05% in 2018 to 2019 and reached 100% in 2020 to 2021. N51I and S108N remained at high levels between 2016 and 2021. In terms of haplotypes, there was a low prevalence of the wild-type and single mutant haplotypes in 2016 to 2017 and 2018 to 2019, and both of them disappeared in 2020 to 2021. The prevalence of the triple mutant **I**_51_**R**_59_**N**_108_ increased from 85.59% in 2016 to 2017 to 95.00% in 2020 to 2021 (χ^2^ for trend = 2.591; *P *= 0.107), whereas the prevalence of the double mutant genotype declined in that period (χ^2^ for trend = 4.131; *P* = 0.042). The temporal distribution of polymorphisms in the *Pfdhfr* gene is shown in Table 3 and Fig. 4 and 5.

**TABLE 3:** Temporal distribution of point mutations in *Pfdhfr* from imported P. falciparum isolates

Genotype	No. of isolates with genotype (%)		
	2016–2017 (*n* = 118)	2018–2019 (*n* = 84)	2020–2021 (*n* = 20)
SNPs			
N51I	112 (94.92)	80 (95.24)	19 (95.00)
C59R	106 (89.93)	79 (94.05)	20 (100.00)
S108N	117 (99.15)	83 (98.81)	20 (100.00)
Haplotypes^*a*^			
N_51_C_59_S_108_	1 (0.85)	1 (1.19)	0
N_51_C_59_**N**_108_	0	1 (1.19)	0
**I**_51_C_59_**N**_108_	11 (9.32)	3 (3.57)	0
N_51_**R**_59_**N**_108_	5 (4.24)	2 (2.38)	1 (5.00)
**I**_51_**R**_59_**N**_108_	101 (85.59)	77 (91.67)	19 (95.00)

a Boldface type indicates the mutant amino acid.

### Spatial distribution of SNPs and haplotypes of *Pfdhps*.

**FIG 6:**
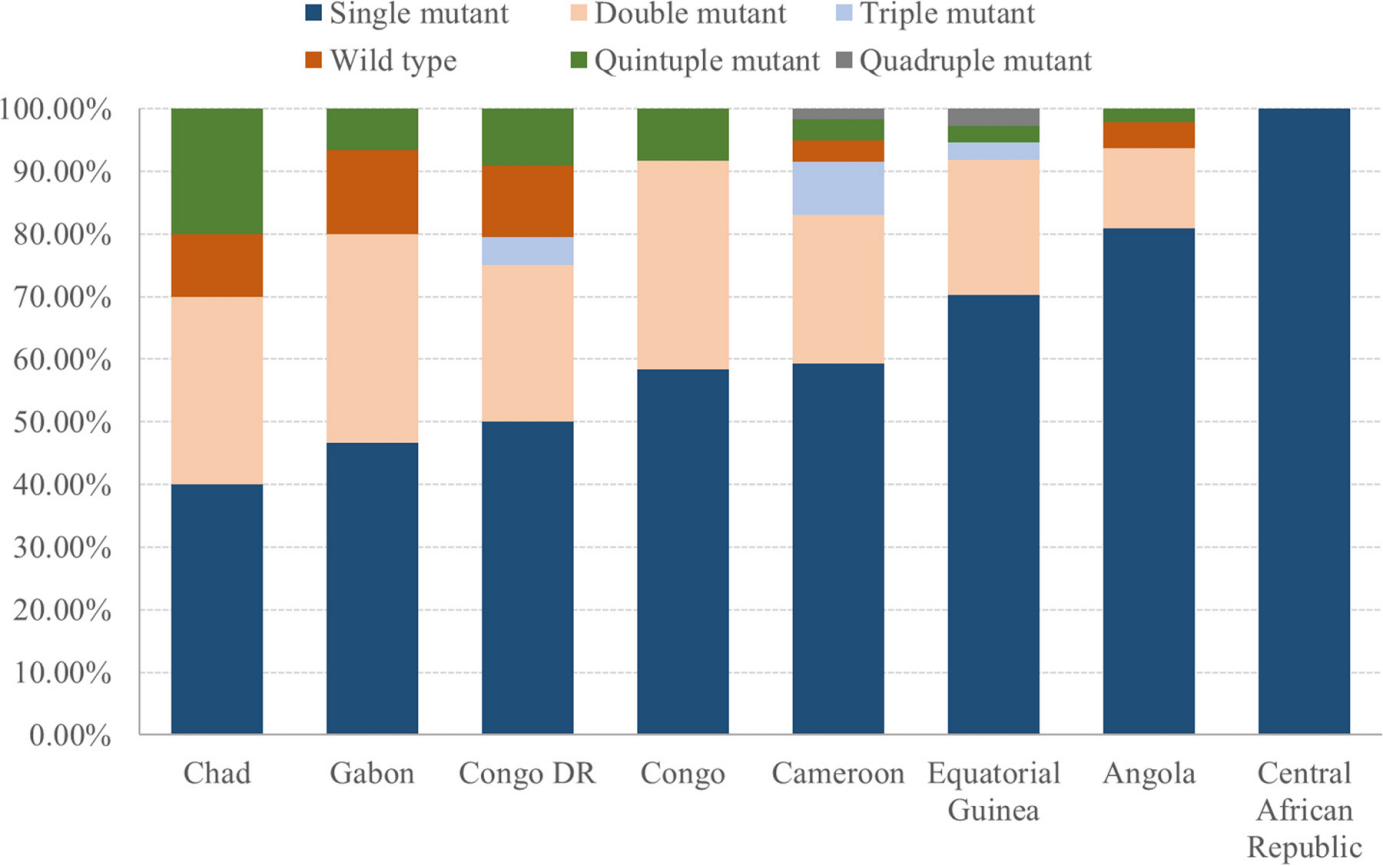
Spatial distribution of *Pfdhps* genotypes in P. falciparum cases imported from central African countries between 2016 and 2021.

Of the 237 parasite-positive samples, 228 samples were successfully genotyped for the *Pfdhps* gene. The prevalences and distributions of SNPs and haplotypes are summarized in Table 4. The point mutation A437G was the most common, occurring in 83.33% (190/228) of the isolates, followed by S436A (28.51%; 65/228). High proportions of the mutations I431V (7.89%; 18/228) and K540E (12.28%; 28/228) were also detected. A581G and A613S were present in only 8.33% (19/228) and 7.89% (18/228) of the samples, respectively. The profiles of SNPs differed between countries. Specifically, A437G predominated in Cameroon (81.36%; 48/59), Angola (91.49%; 43/47), Congo DR (77.27%; 34/44), Equatorial Guinea (97.30%; 36/37), Congo (86.67%; 13/15), and Gabon (91.67%; 11/12) relative to the other SNPs, whereas S436A was more common in Chad (80.00%; 8/10) and the Central African Republic (100.00%; 4/4). Fifteen distinct haplotypes were detected in the study area, among which the single mutant I_431_S_436_**G**_437_K_540_A_581_A_613_ (51.75%; 118/228) was the one most frequently seen. Furthermore, 25 (10.96%) and 24 (10.53%) samples contained the double mutant I_431_S_436_**G**_437_**E**_540_A_581_A_613_ and the single mutant I_431_**A**_436_A_437_K_540_A_581_A_613_, respectively. The patterns of haplotypes varied regionally. The single mutant I_431_S_436_**G**_437_K_540_A_581_A_613_ was the most common type in Cameroon (47.46%; 28/59), Angola (74.47%; 35/47), Congo DR (38.64%; 17/44), Equatorial Guinea (67.57%; 25/37), Gabon (46.67%;7/15), and Congo (50.00%; 6/12), whereas Chad (40.00%; 4/10) and the Central African Republic (100.00%; 4/4) had higher proportions of the single mutation type I_431_**A**_436_A_437_K_540_A_581_A_613_. The spatial distribution of the *Pfdhps* genotypes is shown in Fig. 6.

**TABLE 4:** Polymorphisms observed in *Pfdhps* from P. falciparum isolates imported from central Africa

Genotype	No. of isolates with *Pfdhps* polymorphism (%)								
	Cameroon (*n* = 59)	Angola (*n* = 47)	Congo DR (*n* = 44)	Equatorial Guinea (*n* = 37)	Gabon (*n* = 15)	Congo (*n* = 12)	Chad (*n* = 10)	Central African Republic (*n* = 4)	Total
SNPs									
I431V	5 (8.47)	1 (2.13)	4 (9.09)	4 (10.81)	1 (6.67)	1 (8.33)	2 (20.00)	0	18 (7.89)
S436A	25 (42.37)	6 (12.77)	9 (20.45)	6 (16.22)	3 (20.00)	4 (33.33)	8 (80.00)	4 (100.00)	65 (28.51)
A437G	48 (81.36)	43 (91.49)	34 (77.27)	36 (97.30)	13 (86.67)	11 (91.67)	5 (50.00)	0	190 (83.33)
K540E	4 (6.78)	3 (6.38)	12 (27.27)	4 (10.81)	2 (13.33)	2 (16.67)	1 (10.00)	0	28 (12.28)
A581G	3 (5.08)	2 (4.26)	6 (13.64)	2 (5.41)	1 (6.67)	1 (8.33)	2 (20.00)	0	17 (7.46)
A613S	5 (8.47)	1 (2.13)	5 (11.36)	2 (5.41)	2 (13.33)	1 (8.33)	2 (20.00)	0	18 (7.89)
Haplotypes^*a*^									
Wild type									
I_431_S_436_A_437_K_540_A_581_A_613_	2 (3.39)	2 (4.26)	5 (11.36)	0	2 (13.33)	0	1 (10.00)	0	12 (5.26)
Single mutant									
I_431_**A**_436_A_437_K_540_A_581_A_613_	9 (15.25)	2 (4.26)	3 (6.82)	1 (2.70)	0	1 (8.33)	4 (40.00)	4 (100.00)	24 (10.53)
I_431_S_436_**G**_437_K_540_A_581_A_613_	28 (47.46)	35 (74.47)	17 (38.64)	25 (67.57)	7 (46.67)	6 (50.00)	0	0	118 (51.75)
I_431_S_436_A_437_K_540_A_581_**S**_613_	0	0	1 (2.27)	0	0	0	0	0	1 (0.44)
I_431_S_436_A_437_**E**_540_A_581_A_613_	0	0	1 (2.27)	0	0	0	0	0	1 (0.44)
Double mutant									
I_431_**A**_436_**G**_437_K_540_A_581_A_613_	8 (13.56)	3 (6.38)	2 (4.55)	3 (8.11)	2 (13.33)	2 (16.67)	2 (20.00)	0	22 (9.65)
I_431_S_436_**G**_437_**E**_540_A_581_A_613_	4 (6.78)	3 (6.38)	9 (20.45)	4 (10.81)	2 (13.33)	2 (16.67)	1 (10.00)	0	25 (10.96)
I_431_S_436_**G**_437_K_540_A_581_**S**_613_	0	0	0	0	1 (6.67)	0	0	0	1 (0.44)
**V**_431_S_436_**G**_437_K_540_A_581_A_613_	0	0	0	1 (2.70)	0	0	0	0	1 (0.44)
I_431_S_436_**G**_437_K_540_**G**_581_A_613_	0	1 (2.13)	0	0	0	0	0	0	1 (0.44)
Triple mutant									
I_431_**A**_436_**G**_437_K_540_A_581_**S**_613_	2 (3.39)	0	0	0	0	0	0	0	2 (0.88)
I_431_S_436_**G**_437_**E**_540_**G**_581_A_613_	0	0	2 (4.55)	0	0	0	0	0	2 (0.88)
**V**_431_**A**_436_**G**_437_K_540_A_581_A_613_	3 (5.08)	0	0	1 (2.70)	0	0	0	0	4 (1.75)
Quadruple mutant									
I_431_**A**_436_**G**_437_K_540_**G**_581_**S**_613_	1 (1.69)	0	0	0	0	0	0	0	1 (0.44)
**V**_431_S_436_**G**_437_K_540_**G**_581_**S**_613_	0	0	0	1 (2.70)	0	0	0	0	1 (0.44)
Quintuple mutant									
**V**_431_**A**_436_**G**_437_K_540_**G**_581_**S**_613_	2 (3.39)	1 (2.13)	4 (9.09)	1 (2.70)	1 (6.67)	1 (8.33)	2 (20.00)	0	12 (5.26)

a Boldface type indicates the mutant amino acid.

### Temporal distribution of SNPs and haplotypes of *Pfdhps*.

A comparison of the prevalences of SNPs between groups showed a reduction in the prevalence of I431V with time (χ^2^ for trend = 4.584; *P* = 0.032). Slight reductions in A437G, K540E, and A581G and small increases in S436A and A613S were also detected; however, there were no statistically significant fluctuations in the proportions of these SNPs between 2016 and 2021. Regarding the *Pfdhps* haplotypes, the frequencies of the wild type and triple mutant type declined, whereas the prevalence of the single mutant haplotype increased from 61.60% in 2016 to 2017 to 73.68% in 2020 to 2021 (χ^2^ for trend = 0.704; *P* = 0.401). The temporal distribution of the polymorphisms of the *Pfdhps* gene is shown in Table 5 and Fig. 4 and 5.

**TABLE 5:** Temporal distribution of point mutations in *Pfdhps* in imported P. falciparum isolates

Genotype	No. of isolates (%)		
	2016–2017 (*n* = 125)	2018–2019 (*n* = 84)	2020–2021 (*n* = 19)
SNPs			
I431V	14 (11.20)	4 (4.76)	0
S436A	36 (28.80)	23 (27.38)	6 (31.58)
A437G	107 (85.60)	68 (80.95)	15 (78.95)
K540E	16 (12.80)	10 (11.90)	2 (10.53)
A581G	11 (8.80)	5 (5.95)	1 (5.26)
A613S	11 (8.80)	5 (5.95)	2 (10.53)
Haplotypes			
Wild type	5 (4.00)	7 (8.33)	0
Single mutation	77 (61.60)	53 (63.10)	14 (73.68)
Double mutation	28 (22.40)	18 (21.43)	4 (21.05)
Triple mutation	6 (4.80)	2 (2.38)	0
Quadruple mutation	1 (0.80)	0	1 (5.26)
Quintuple mutation	8 (6.40)	4 (4.76)	0

### Patterns and prevalence of *Pfdhfr-Pfdhps*.

The patterns of the *Pfdhfr-Pfdhps* combination were analyzed, as summarized in Tables 6 and 7. Twenty-six haplotypes were identified, but only one sample was of the wild type. The prevalence of the quadruple mutant **I**_51_**R**_59_**N**_108_-I_431_S_436_**G**_437_K_540_A_581_A_613_ was the highest (45.45%; 100/220), followed by the quadruple mutant **I**_51_**R**_59_**N**_108_-I_431_**A**_436_A_437_K_540_A_581_A_613_ (10.00%; 22/220) and the quintuple mutant **I**_51_**R**_59_**N**_108_-I_431_**A**_436_**G**_437_K_540_A_581_A_613_ (9.09%; 20/220). The combination of mutant *Pfdhfr* and *Pfdhps* genes conferring partial resistance to SP was detected in 140 samples (63.64%; 140/220) (Table 7). The combination of mutant *Pfdhfr* and *Pfdhps* genes conferring full resistance to SP was detected in 8.64% (19/220) of the isolates, including 7 from Congo DR, 4 from Cameroon, 3 from Equatorial Guinea, 2 from Angola, 2 from Gabon, and 1 from Congo. Alarmingly, the superresistant *Pfdhfr-Pfdhps* haplotype, combining N51I, N59R, and S108N in *Pfdhfr* and A437G, K540E, and A581G in *Pfdhps*, was observed in two isolates from Congo DR in 2016 and 2018. Furthermore, an octuple *Pfdhfr*-*Pfdhps* allele (**I**_51_**R**_59_**N**_108_-**V**_431_**A**_436_**G**_437_K_540_**G**_581_**S**_613_) was seen in 5.00% (11/220) of samples from seven of the eight central African countries from which the samples were drawn (3 samples from Congo DR, 2 from Cameroon, 2 from Chad, and 1 each from Angola, Congo, Gabon, and Equatorial Guinea).

**TABLE 6:** Prevalence of *Pfdhfr-Pfdhps* haplotypes in P. falciparum isolates imported from central Africa

Haplotype	Codon^*d*^	No. of isolates (%)
Wild type	N_51_C_59_S_108_-I_431_S_436_A_437_K_540_A_581_A_613_	1 (0.45)

Single mutant	N_51_C_59_S_108_-I_431_S_436_**G**_437_K_540_A_581_A_613_	1 (0.45)

Double mutant	**I**_51_C_59_**N**_108_-I_431_S_436_A_437_K_540_A_581_A_613_	1 (0.45)

Triple mutant	N_51_C_59_**N**_108_-I_431_S_436_**G**_437_**E**_540_A_581_A_613_	1 (0.45)
	**I**_51_C_59_**N**_108_-I_431_**A**_436_A_437_K_540_A_581_A_613_	1 (0.45)
	**I**_51_C_59_**N**_108_-I_431_S_436_**G**_437_K_540_A_581_A_613_	10 (4.55)
	**I**_51_**R**_59_**N**_108_-I_431_S_436_A_437_K_540_A_581_A_613_	10 (4.55)
	N_51_**R**_59_**N**_108_-I_431_S_436_**G**_437_K_540_A_581_A_613_	3 (1.36)

Quadruple mutant	**I**_51_**R**_59_**N**_108_-I_431_**A**_436_A_437_K_540_A_581_A_613_	22 (10.00)
	**I**_51_**R**_59_**N**_108_-I_431_S_436_A_437_K_540_A_581_**S**_613_	1 (0.45)
	**I**_51_**R**_59_**N**_108_-I_431_S_436_**G**_437_K_540_A_581_A_613_^*a*^	100 (45.45)
	N_51_**R**_59_**N**_108_-I_431_**A**_436_**G**_437_K_540_A_581_A_613_	2 (0.91)
	N_51_**R**_59_**N**_108_-I_431_S_436_**G**_437_**E**_540_A_581_A_613_	3 (1.36)
	**I**_51_**R**_59_**N**_108_-I_431_S_436_A_437_**E**_540_A_581_A_613_	1 (0.45)
	**I**_51_C_59_**N**_108_-I_431_S_436_**G**_437_**E**_540_A_581_A_613_	1 (0.45)

Quintuple mutant	**I**_51_**R**_59_**N**_108_-I_431_**A**_436_**G**_437_K_540_A_581_A_613_^*a*^	20 (9.09)
	**I**_51_**R**_59_**N**_108_-I_431_S_436_**G**_437_**E**_540_A_581_A_613_^*b*^	19 (8.64)
	**I**_51_**R**_59_**N**_108_-I_431_S_436_**G**_437_K_540_**G**_581_A_613_^*a*^	1 (0.45)
	**I**_51_**R**_59_**N**_108_-**V**_431_S_436_**G**_437_K_540_A_581_A_613_^*a*^	1 (0.45)

Sextuple mutant	**I**_51_**R**_59_**N**_108_-I_431_S_436_**G**_437_**E**_540_**G**_581_A_613_^*c*^	2 (0.91)
	**I**_51_**R**_59_**N**_108_-I_431_**A**_436_**G**_437_K_540_A_581_**S**_613_^*a*^	2 (0.91)
	**I**_51_**R**_59_**N**_108_-**V**_431_**A**_436_**G**_437_K_540_A_581_A_613_^*a*^	3 (1.36)

Septuple mutant	**I**_51_C_59_**N**_108_-**V**_431_**A**_436_**G**_437_K_540_**G**_581_**S**_613_	1 (0.45)
	**I**_51_**R**_59_**N**_108_-I_431_**A**_436_**G**_437_K_540_**G**_581_**S**_613_^*a*^	1 (0.45)
	**I**_51_**R**_59_**N**_108_-**V**_431_S_436_**G**_437_K_540_**G**_581_**S**_613_^*a*^	1 (0.45)

Octuple mutant	**I**_51_**R**_59_**N**_108_-**V**_431_**A**_436_**G**_437_K_540_**G**_581_**S**_613_^*a*^	11 (5.00)

Total		220 (100.00)

a Partially resistant mutation.

b Fully resistant mutation.

c Superresistant mutation.

d Boldface type indicates the mutant amino acid.

**TABLE 7:** Prevalences of partially resistant, fully resistant, and superresistant mutations of *Pfdhfr-Pfdhps* haplotypes by country

Haplotype^*a*^	No. of isolates (%)								
	Cameroon (*n* = 58)	Angola (*n* = 44)	Congo DR (*n* = 42)	Equatorial Guinea (*n* = 36)	Gabon (*n* = 14)	Congo (*n* = 12)	Chad (*n* = 10)	Central African Republic (*n* = 4)	Total
Partially resistant									
**I**_51_**R**_59_**N**_108_-I_431_**A**_436_**G**_437_K_540_A_581_A_613_	8 (13.79)	2 (4.55)	2 (4.76)	3 (8.33)	2 (14.29)	2 (16.67)	1 (10.00)	0	20 (9.09)
**I**_51_**R**_59_**N**_108_-I_431_**A**_436_**G**_437_K_540_A_581_**S**_613_	2 (3.45)	0	0	0	0	0	0	0	2 (0.91)
**I**_51_**R**_59_**N**_108_-I_431_**A**_436_**G**_437_K_540_**G**_581_**S**_613_	1 (1.72)	0	0	0	0	0	0	0	1 (0.45)
**I**_51_**R**_59_**N**_108_-I_431_S_436_**G**_437_K_540_**G**_581_A_613_	0	1 (2.27)	0	0	0	0	0	0	1 (0.45)
**I**_51_**R**_59_**N**_108_-I_431_S_436_**G**_437_K_540_A_581_A_613_	26 (44.83)	23 (52.27)	14 (33.33)	25 (69.44)	7 (50.00)	5 (41.67)	0	0	100 (45.45)
**I**_51_**R**_59_**N**_108_-**V**_431_**A**_436_**G**_437_K_540_A_581_A_613_	2 (3.45)	0	0	1 (2.78)	0	0	0	0	3 (1.36)
**I**_51_**R**_59_**N**_108_-**V**_431_**A**_436_**G**_437_K_540_**G**_581_**S**_613_	2 (3.45)	1 (2.27)	3 (7.14)	1 (2.78)	1 (7.14)	1 (8.33)	2 (20.00)	0	11 (5.00)
**I**_51_**R**_59_**N**_108_-**V**_431_S_436_**G**_437_K_540_A_581_A_613_	0	0	0	1 (2.78)	0	0	0	0	1 (0.45)
**I**_51_**R**_59_**N**_108_-**V**_431_S_436_**G**_437_K_540_**G**_581_**S**_613_	0	0	0	1 (2.78)	0	0	0	0	1 (0.45)

Subtotal	41 (70.69)	27 (61.36)	19 (45.24)	32 (88.89)	10 (71.43)	8 (66.67)	3 (30.00)	0	140 (63.64)
Fully resistant									
**I**_51_**R**_59_**N**_108_-I_431_S_436_**G**_437_**E**_540_A_581_A_613_	4 (6.90)	2 (4.55)	7 (16.67)	3 (8.33)	2 (14.29)	1 (8.33)	0	0	19 (8.64)
Superresistant									
**I**_51_**R**_59_**N**_108_-I_431_S_436_**G**_437_**E**_540_**G**_581_A_613_	0	0	2 (4.76)	0	0	0	0	0	2 (0.91)

a Boldface type indicates the mutant amino acid.

## DISCUSSION

Monitoring the dynamics of the mutations in the *Pfdhfr* and *Pfdhps* genes that confer SP resistance is critical, especially in those African countries where SP is recommended as an IPTp or IPTi. In this study, we focused on central Africa, which accounts for about 25% of the malaria burden in the WHO African region. Although the prevalence of mutant *Pfdhfr* or *Pfdhps* genes in central Africa was lower than that in western Africa at the regional level (18, 19), we found that molecular markers of SP resistance in the *Pfdhfr* gene occurred at high frequencies in parasites sampled from central Africa, confirming the selection for resistance to pyrimethamine. At the country level, point mutations at residues 51, 59, and 108 in *Pfdhfr* were highly prevalent in all eight countries of central Africa from which malaria was imported in the present study. These mutations have also been observed at high levels in neighboring countries, including Nigeria (18), Kenya (20), and Rwanda (21). Compared with published data from October 2005 and February 2011, the prevalence of N51I, C59R, and S108N in Gabon and Equatorial Guinea has increased dramatically from <50% to 100% in this survey (17). These data also show that the *Pfdhfr* triple mutant **I**_51_**R**_59_**N**_108_ was dominant and much more common (~71.11% to 100%) than single (N_51_C_59_**N**_108_) or double (**I**_51_C_59_**N**_108_-N_51_**R**_59_**N**_108_) mutants between 2016 and 2021. This distribution of *Pfdhfr* haplotypes was similar to those reported previously for the majority of central African countries, such as Cameroon, Congo DR, Congo, Equatorial Guinea, and Gabon (3). However, in the present study, Angola, Cameroon, Congo DR, and Equatorial Guinea showed higher prevalences (71.11%, 96.61%, 88.10%, and 100%, respectively) of **I**_51_**R**_59_**N**_108_ mutants than those in previous reports (15%, 66.4%, 47.9%, and 78%, respectively), indicating the possible increase and spread of pyrimethamine resistance in Angola, Cameroon, Congo DR, and Equatorial Guinea (3, 22–25). Our analysis of the temporal trends in SP resistance reveals an increased frequency of **I**_51_**R**_59_**N**_108_ in the period from 2016 to 2021. It has been reported that the triple mutant **I**_51_**R**_59_**N**_108_ increases the inhibitory constant (*K_i_*) of *Pfdhfr* by >200-fold, whereas the increase in the *K_i_* caused by the single mutant S108N is only 5-fold, and that caused the double mutant C59R,S108N is >50-fold (26). Therefore, the predominance and increasing frequency of the triple mutant **I**_51_**R**_59_**N**_108_ observed in central Africa might impair the efficacy of pyrimethamine, an urgent concern demanding enhanced molecular surveillance.

In this study, A437G was the most prevalent point mutation (83.33%; 190/228) in *Pfdhps* in the majority of the countries in central Africa, except for Chad and the Central African Republic, where there was a higher proportion of the S436A mutation than the A437G mutation. Regarding K540E, the prevalence in Angola (6.38%) from our data was consistent with previously reported results (7%) (22), while the frequency in Congo DR (27.27%) was lower than those in previous reports (~32.6% to >50%) (27, 28). These findings demonstrate the spatial heterogeneity of *Pfdhps* point mutations in central African countries. The single mutant haplotype I_431_S_436_**G**_437_K_540_A_581_A_613_ was also the most frequently documented *Pfdhps* haplotype in several countries, although the single mutant haplotype I_431_**A**_436_A_437_K_540_A_581_A_613_ predominated in Chad and the Central African Republic. A higher prevalence of highly mutated *Pfdhps* genes (double/triple/quadruple/quintuple mutants) from Congo DR (38.64%) was recorded here than that in a previous study (19%) (29). The high proportion of the single mutant haplotype I_431_S_436_**G**_437_K_540_A_581_A_613_ (51.75%) detected in this study is consistent with or higher than that in previous studies, suggesting an increase in resistance to sulfadoxine in central Africa (3, 22, 23, 30, 31).

Notably, according to our study, the novel mutation I431V, which has been widespread in Nigeria in recent years (32), has emerged in more African countries (23, 33, 34). We detected it in isolates from seven of the eight countries from which malaria was exported, especially in Chad (20.00%), Equatorial Guinea (10.81%), Congo DR (9.09%), Cameroon (8.47%), and Congo (8.33%). Previous reports of this novel mutation (I431V) have speculated its possible effect on the binding efficiency of sulfadoxine, conferring increased resistance to this drug (32). Therefore, the wide dissemination of I431V in central Africa demonstrated here raises urgent concerns, particularly in Chad, Equatorial Guinea, and Congo. To our knowledge, there have been few reports of the distributions of molecular determinants of antimalarial drug resistance in Chad. Our data fill this gap and demonstrate the high prevalence of the S436A (80.00%) and novel I431V (20.00%) mutations and the moderate prevalence of the A437G allele (50.00%) in Chad, warranting enhanced surveillance of sulfadoxine resistance in this country. The frequencies of K540E and A581G remained steady between 2016 and 2021 in the present study. Similarly, a previous model based on the prevalence of K540E and A581G supported stable levels in most countries in central Africa between 2000 and 2020 (35). Nevertheless, we found a trend toward increasing prevalences of S436A and A613S, although they were not statistically significant. Furthermore, the proportion of A581G in our study samples was higher than that in samples obtained from migrant workers returning from central African countries from 2013 to 2016 (36). Taken together, our data indicate a trend toward increasing sulfadoxine resistance in central Africa.

It is worrisome that the triple mutation I_431_S_436_**G**_437_**E**_540_**G**_581_A_613_ in *Pfdhps*, which was previously reported in Rwanda and Tanzania (3, 37), was identified in two isolates from Congo DR. Moreover, the double mutation I_431_S_436_**G**_437_**E**_540_A_581_A_613_ in *Pfdhps* was widely detected in all countries except the Central African Republic. A437G and K540E are the key mutations conferring sulfadoxine resistance, and structural analysis revealed that their combination results in an additive effect of ~117-fold (38). A437G and K540E were strongly associated with SP treatment failure in therapeutic efficacy trials (39). Therefore, when mutations in *Pfdhfr* and *Pfdhps* are combined, the quintuple mutant **I**_51_**R**_59_**N**_108_-**G**_437_**E**_540_ and the sextuple mutant **I**_51_**R**_59_**N**_108_-**G**_437_**E**_540_**G**_581_ are classified as fully resistant and superresistant haplotypes, respectively (17). Our analysis of the patterns of *Pfdhfr*-*Pfdhps* alleles showed the frequent occurrence of the partially resistant combination (63.64%) in central Africa, whereas the fully resistant combination was detected in 8.64% of isolates. It is inappropriate to compare the prevalences of *Pfdhfr*-*Pfdhps* alleles directly with those in other studies because of differences in participants, sampling areas, and years.

Our data also confirm the continued emergence of superresistant *Pfdhfr*-*Pfdhps* alleles in Congo DR, which was previously studied in isolates from this country sampled between 2014 and 2015 (6). Strikingly, seven of the eight countries from which malaria was imported reported the octuple *Pfdhfr*-*Pfdhps* mutant **I**_51_**R**_59_**N**_108_-**V**_431_**A**_436_**G**_437_K_540_**G**_581_**S**_613_, with a total prevalence of 5.00%. Although it did not occur together with the K540E substitution, the presence of this octuple mutant reinforces our concern for the wide dissemination and high level of SP resistance in central Africa, which may limit the efficacy of IPTp.

This study was limited by its small sample sizes from the northern countries of central Africa, especially from 2020 to 2021, which may have resulted from the passage of fewer migrant workers in response to the COVID-19 pandemic. The prevalence of *Pfdhfr* and *Pfdhps* mutations may be a rough estimate or an underestimate because of the limited sample size, particularly in Gabon, Congo, Chad, and the Central African Republic. Furthermore, specific sampling site information for the isolates was not collected, which limited the interpretation of the spatial distribution of *Pfdhfr*-*Pfdhps* mutations in each country. Thus, additional samples from the northern countries of central Africa are recommended for future studies. The origins of SP resistance lineages between countries or regions of Africa should also be further investigated.

### Conclusion.

In this study, we demonstrated the wide geographic spread and the increasing trend of highly resistant *Pfdhfr* genes in central Africa between 2016 and 2021. The profiles of mutant *Pfdhps* genes showed varied spatial patterns between countries, with S436A predominating in northern countries and A437G predominating in the other countries. This study also demonstrated the wide dissemination of the novel mutant *Pfdhps* I431V and the high prevalence of partially resistant, fully resistant, and superresistant *Pfdhfr*-*Pfdhps* combinations, prompting urgent concern for the efficacy of SP in central Africa. These observations are alarming in central African countries where malaria is endemic, where SP is widely used for IPTp and IPTi, and we recommend that molecular surveillance be enhanced to continuously monitor the status of the *Pfdhfr* and *Pfdhps* genes in these territories.

## MATERIALS AND METHODS

### Study site and design.

An observational study was undertaken. All cases of imported P. falciparum infection in patients returning to Zhejiang Province from central African counties between 2016 and 2021 were investigated, and samples were collected. Zhejiang Province is located in eastern China. Many laborers and businessmen travel frequently between Zhejiang Province and central African countries. Therefore, cases of malaria imported from these central African countries are frequently seen.

In this study, individuals with malaria-related symptoms and positive results by microscopic assessment or rapid diagnostic tests were reported by hospitals or clinics. The local Centers for Diseases Control and Prevention (CDC) then performed epidemiological analyses and laboratory tests by microscopy. The Zhejiang Provincial CDC also double-checked the diagnosis and confirmed the parasite species with both thick and thin blood smears and PCR.

### Sample collection and DNA extraction.

A total of 237 P. falciparum infections imported from central African countries to Zhejiang Province, China, between January 2016 and December 2021 were investigated. Approximately 1 mL of venous blood was collected from each patient before antimalarial treatment. All blood samples were stored at −80°C until analysis. The genomic DNA was extracted with the QIAamp DNA minikit (Qiagen Inc., Germany), according to the manufacturer’s instructions.

### DNA amplification and sequencing.

Molecular markers (mutations) in the *Pfdhfr* and *Pfdhps* genes, which are associated with drug resistance in P. falciparum isolates, were detected by nested PCR, as previously described (40–42). Sequences encoding amino acid positions 50, 51, 59, 108, 139, and 164 of *Pfdhfr* and positions 431, 436, 437, 540, 581, and 613 of *Pfdhps* were amplified. The primers and cycling conditions for the *Pfdhfr* and *Pfdhps* genes were described previously (42).

### Data analysis.

MEGA version 7.0.26 (https://www.megasoftware.net/) was used to align the amplicon sequences with reference sequences retrieved from the National Center for Biotechnology Information database. The GenBank accession numbers of the reference sequences are NC_004318.2 for *Pfdhfr* and XM_001349382.1 for *Pfdhps*. A database was constructed with Microsoft Excel 2017. A map of the numbers of cases from each country was generated with ArcGIS 10.1 software (http://www.esri.com/arcgis/about-arcgis). To analyze the temporal distribution of the point mutations and haplotypes of *Pfdhfr* and *Pfdhps*, the isolates were classified into three groups according to the year of sampling (2016 to 2017, 2018 to 2019, and 2020 to 2021). The sample sizes of the three groups were 129, 85, and 23, respectively. The Mantel-Haenszel χ^2^ test was used to evaluate the differences in the prevalences of single-nucleotide polymorphisms (SNPs) and haplotypes among these three groups to examine the temporal trends in *Pfdhfr* and *Pfdhps* mutations. All statistical analyses were performed with IBM SPSS Statistics for Windows, version 21.0 (IBM Corp., Armonk, NY, USA). Variables with a *P* value of <0.05 were considered statistically significant.

### Ethics statement.

This study was approved by the Ethical Review Committee of the Zhejiang Center for Disease Control and Prevention.

### Data availability.

The original contributions presented in this study are included in the article; further inquiries can be directed to the corresponding authors.
